# Synthesis, Characterization and Antitumour Activity of Di-n-Butyltin Salicyloxamate

**DOI:** 10.1155/MBD.1998.265

**Published:** 1998

**Authors:** Marcel Gielen, Hassan Dalil, Dick de Vos, Monique Biesemans, Rudolph Willem

**Affiliations:** 1 Vrije Universiteit Brussel Pleinlaan 2 Brussels B-1050 Belgium; 2 Vrije Universiteit Brussel Department of General and Organic Chemistry Faculty of Applied Sciences Pleinlaan 2 Brussels B-1050 Belgium; 3 Medical Department Pharmachemie B. V. Haarlem NL-2003 RN The Netherlands


### SYNTHESIS, CHARACTERIZATION AND ANTITUMOUR ACTIVITY OF DI-n-BUTYLTIN SALICYLOXAMATE

Marcel Gielen*[1a], Hassan Dalil[1a],

Dick de Vos[2], Monique Biesemans[1], and Rudolph Willem[1]

[1] Vrije Universiteit Brussel, Pleinlaan 2, B-1050 Brussels, Belgium
[a] Department of General and Organic Chemistry, Faculty of Applied Sciences; [b] High Resolution NMR Centre
[2] Medical Department, Pharmachemie B. V., NL-2003 RN Haarlem, the Netherlands

**Abstract**
The synthesis and characterization of di-n-butyltin and dimethyltin salicyloxamate, respectively compounds **1** and **2**, are reported. Compound **1** is more active than cisplatin, 5-fluorouracil and etoposide against seven tumoural cell lines of human origin, but less active than methotrexate and doxorubucin.

Diorganotin derivatives of salicylic acid and its substituted analogs exhibit antitumour activities *in vitro* against human tumoural cell lines[1,2]. The phenolic hydroxy group of salicylic acid is not involved in its reaction with diorganotin oxides[3,4]. In fact, salicylic acid behaves in this respect as benzoic acid, giving rise to diorganotin disalicylates with free hydroxy groups.
This report presents the synthesis and characterization of di-n-butyltin and dimethyltin salicyloxamate, respectively compounds **1** and **2**, in order to find out whether the phenolic hydroxy group of salicyloxamic acid is involved when reacting with diorganotin oxides. The antitumour activity of compound **1** was determined for comparison with that of the corresponding di-n-butyltin salicylate[5].

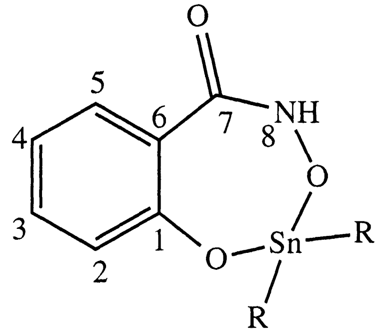

| | α β γ δ | |
|---|---|---|
| R = | $CH_2$-$CH_2$-$CH_2$-$CH_3$ | **1** |
| | α | |
| | $CH_3$ | **2** |

Compound **1**, a viscous oil, and compound **2**, a solid (m.p. 195-197°) were synthesized with 94% and 85% yield, respectively, by condensing the appropriate diorganotin oxide with salicyloxamic acid in a toluene/ethanol 4/1 mixture[3,6,7]. They were purified by chromatography on Sephadex LH-20 with chloroform/ethanol 99/1 as eluent (compound **1**) or crystallization from dichloromethane/hexane 50/50 (compound **2**). $^{1}$H and $^{13}$C NMR data ($CDCl_3$) are given in Table 1. The $^{117}$Sn NMR chemical shifts of compounds **1** and **2** are respectively -140.0 and -100.2 ppm. The cationic mode electrospray mass spectra of compounds **1** and **2** exhibit the fragment corresponding to (M + $H^+$) (100%) at resp. m/z = 386 ($C_{15}H_{24}NO_3Sn$) and 302 ($C_9H_{12}NO_3Sn$) with the expected isotopic distribution.
Compound **1** was tested *in vitro* against seven tumoural cell lines of human origin: MCF-7 et EVSA-T, two mammary tumours, WiDr, a colon carcinoma, IGROV, an ovarian cancer, M19 MEL, a melanoma, A498, a renal cancer, and H226, a non-small cell lung cancer, as water/ethanol 99/1 solutions.
The results of the antitumoural tests are summarized in Table 2 and compared with the inhibition doses $ID_{50}$ found against MCF-7 and WiDr for di-n-butyltin disalicylate and bis(acetylsalicylate), as well as those obtained for some clinically used reference compounds[8,9], cis-platin, 5-fluorouracil, etoposide, methotrexate and doxorubicin. Compound **1** has, especially against WiDr, a markedly higher *in vitro* activity than di-n-butyltin disalicylate and bis(acetylsalicylate) as well as cisplatin, 5-fluorouracil and etoposide. Its activity is lower than that of methotrexate and doxorubicin, except for H-226 against which compound **1** exhibits a very low inhibition dose as compared to all other compounds.

| | $^1H$ | | | $^{13}C$ | |
|---|---|---|---|---|---|
| | **1** | **2** | | **1** | **2** |
| | | | $C_1$ | 162.7 | 162.1 |
| $H_2$ | d: 6.54 [8] | d: 6.55 [8] | $C_2$ | 119.5 | 119.6 |
| $H_3$ | dd: 6.79 [8,8] | dd: 6.80 [8,8] | $C_3$ | 116.8 | 116.2 |
| $H_4$ | dd: 7.28 [8,8] | dd: 7.30 [8,8] | $C_4$ | 133.0 | 133.1 |
| $H_5$ | d: 7.88 [8] | d: 7.90 [8] | $C_5$ | 129.6 | 129.6 |
| | | | $C_6$ | 116.3 | 116.2 |
| | | | $C_7$ | 163.3 | 163.4 |
| $H_8$ | s: 13.62 (**55**) | s: 13.60 (**55**) | | | |
| $H_\alpha$ | - | s: 0.84 (**76/73**) | $C_\alpha$ | 22.7 (**590/564**) | 2.1 (**639/611**) |
| $H_{\alpha\text{-}\beta}$ | m: 1.75-1.52 | - | $C_\beta$ | 26.8 (**31**) | - |
| $H_\gamma$ | tq: 1.37 [7,7] | - | $C_\gamma$ | 26.6 (**88**) | - |
| $H_\delta$ | t: 0.87 [7] | - | $C_\delta$ | 13.6 | - |

Table 1: $^1H$ and $^{13}C$ NMR data of compounds **1** and **2** ($CDCl_3$). Chemical shifts in ppm vs. TMS; $^nJ(^1H,^1H)$ (between brackets), $^nJ(^1H,^{119/117}Sn)$ and $^nJ(^{13}C,^{119/117}Sn)$ (bold in parentheses) coupling constants in Hz. d: doublet; t: triplet; tq: triplet of quartets; s: singlet; m: complex pattern.

| | MCF-7 | EVSA-T | WiDr | IGROV | M19 MEL | A498 | H226 |
|---|---|---|---|---|---|---|---|
| **1** | 67 | 59 | 316 | 103 | 90 | 140 | 109 |
| Di-n-butyltin disalicylate | 540 | - | 2974 | - | - | - | - |
| Di-n-butyltin bis(acetylsalicylate) | 283 | - | 2495 | - | - | - | - |
| Cisplatin | 699 | 422 | 967 | 169 | 558 | 2253 | 3269 |
| 5-Fluorouracil | 750 | 475 | 225 | 297 | 442 | 143 | 340 |
| Etoposide | 2594 | 317 | 150 | 580 | 505 | 1314 | 3934 |
| Methotrexate | 18 | 5 | <3 | 7 | 23 | 37 | 2287 |
| Doxorubicin | 10 | 8 | 11 | 60 | 16 | 90 | 199 |

Table 2. Inhibition doses $ID_{50}$ of compound **1** as compared to di-n-butyltin disalicylate and bis(acetylsalicylate)[5] and of some reference compounds[8,9] against tumoural cell lines of human origin.

**Acknowledgements**

We thank Mrs. I. Verbruggen for recording the NMR spectra. We are grateful to Mr. H. J. Kolker, Dr. J. Verweij, Prof. Dr. G. Stoter, Dr. J. H. M. Schellens, Laboratory of Experimental Chemotherapy and Pharmacology, Department of Medical Oncology, Rotterdam Cancer Institute, NL - 3008 AE, Rotterdam, The Netherlands, for performing the *in vitro* tests. This research was supported by the Belgian "Nationaal Fonds voor Wetenschappelijk Onderzoek" (N.F.W.O. grant nr G.0054.96, M. G.), and the Fund for Scientific Research Flanders (Belgium, grant nr G.0192.98, R. W., M. B.).

**References**

1. M. Gielen, P. Lelieveld, D. de Vos, R. Willem, "Metal Complexes in Cancer Chemotherapy", chapter 17 , ed. B.K. Keppler, VCH, Weinheim, 1993, pp. 383 - 390.
2. M. Gielen, *Coord. Chem. Rev.*, **151** (1996), 41.
3. M. Bouâlam, R. Willem, M. Biesemans, B. Mahieu, J. Meunier-Piret, M. Gielen, *Main Group Met. Chem.*, **14** (1991), 41.
4. M. Bouâlam, R. Willem, M. Biesemans, M. Gielen, *Appl. Organomet. Chem.*, **5** (1991), 497.
5. A. Meriem, R. Willem, M. Biesemans, B. Mahieu, D. de Vos, P. Lelieveld, M. Gielen, *Appl. Organomet. Chem.*, **5** (1991), 195.
6. M. Gielen, A. El Khloufi, M. Biesemans, B. Mahieu, R. Willem, *Bull. Soc. Chim. Belg.*, **101** (1992), 243.
7. M. Bouâlam, R. Willem, M. Biesemans, M. Gielen, *Appl. Organomet. Chem.*, **5** (1991), 497.
8. P. Skehan, R. Storeng, D. Scudiero, A. Monks, J. McMahon, D. Vistica, J. T. Warren, H. Bokesch, S. Kenney, M. R. Boyd, *J. Natl. Cancer Inst.*, **82** (1990), 1107.
9. Y. P. Kepers, G. J. Peters, J. Van Ark-Otte, B. Winograd, H. M. Pinedo, *Eur. J. Cancer*, **27** (1991), 897.

**Received: May 28, 1998 - Accepted: June 24, 1998 -**
**Received in revised camera-ready format: June 25, 1998**

